# Peak oxygen uptake in combination with ventilatory efficiency improve risk stratification in major abdominal surgery

**DOI:** 10.14814/phy2.15904

**Published:** 2024-01-01

**Authors:** Karolina Kristenson, Edvard Gerring, Bergthor Björnsson, Per Sandström, Kristofer Hedman

**Affiliations:** ^1^ Department of Thoracic and Vascular Surgery in Östergötland, and Department of Health, Medicine and Caring Sciences Linköping University Linköping Sweden; ^2^ Department of Clinical Physiology, and Department of Health, Medicine and Caring Sciences Linköping University Linköping Sweden; ^3^ Department of Surgery, Department of Biomedicine and Clinical Sciences Linköping University Linköping Sweden

**Keywords:** abdominal surgery, cardiopulmonary exercise testing, exercise capacity, functional capacity, ventilatory efficiency

## Abstract

This pilot study aimed to evaluate if peak VO_2_ and ventilatory efficiency in combination would improve preoperative risk stratification beyond only relying on peak VO_2_. This was a single‐center retrospective cohort study including all patients who underwent cardiopulmonary exercise testing (CPET) as part of preoperative risk evaluation before major upper abdominal surgery during years 2008–2021. The primary outcome was any major cardiopulmonary complication during hospitalization. Forty‐nine patients had a preoperative CPET before decision to pursue to surgery (cancer in esophagus [*n* = 18], stomach [6], pancreas [16], or liver [9]). Twenty‐five were selected for operation. Patients who suffered any major cardiopulmonary complication had lower ventilatory efficiency (i.e., higher VE/VCO_2_ slope, 37.3 vs. 29.7, *p* = 0.031) compared to those without complications. In patients with a low aerobic capacity (i.e., peak VO_2_ < 20 mL/kg/min) and a VE/VCO_2_ slope ≥ 39, 80% developed a major cardiopulmonary complication. In this pilot study of patients with preoperative CPET before major upper abdominal surgery, patients who experienced a major cardiopulmonary complication had significantly lower ventilatory efficiency compared to those who did not. A low aerobic capacity in combination with low ventilatory efficiency was associated with a very high risk (80%) of having a major cardiopulmonary complication.

## Graphical abstract

In this pilot study of patients who performed CPET before major upper abdominal surgery, we found that patients who suffered a major cardiopulmonary complication had significantly higher (less favorable) values of ventilatory efficiency compared to those who did not sustain a complication. Importantly, we found that by using a combination of low aerobic capacity (peak VO_2_ < 20 mL/kg/min) and ventilatory efficiency (VE/VCO_2_‐slope [greater than or equal to] 39), we were able to identify a group of patients with a particularly high frequency of complications (80%).

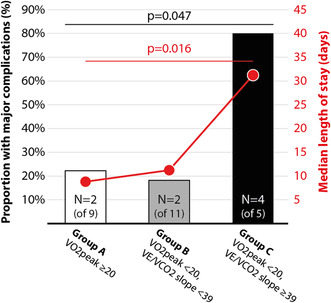

## 1 INTRODUCTION

A strong association exists between cardiorespiratory fitness and surgical outcomes, where fitter patients possess heightened resilience to withstand the stress response imposed by major surgery (Roxburgh et al., 2023). Perioperative cardiovascular guidelines endorse preoperative estimation of functional capacity (Halvorsen et al., 2022), but subjective assessment by the preoperative physician has a low sensitivity in identifying patients with low functional capacity and is an insufficient predictor of postoperative morbidity and mortality (Wijeysundera et al., 2018).

Cardiopulmonary exercise testing (CPET) is the gold standard for objective assessment of exercise tolerance and overall cardiopulmonary function (Levett et al., 2018). Studies support the use of CPET for preoperative risk prediction in esophageal/gastric surgery (Benington et al., 2019; Jack et al., 2014), hepatobiliary surgery (Snowden et al., 2010), and pancreatic surgery (Ausania et al., 2012).

Historically, in abdominal surgery, most studies have used either maximal aerobic capacity (peak VO_2_) with a threshold of 14 mL/kg/min and/or oxygen uptake (VO_2_) at the anaerobic threshold (AT) with a threshold of 11 mL/kg/min to identify patients with a low functional capacity (Wijeysundera et al., 2018). However, advances in CPET methodology and subsequent research have allowed for identification of other measures of relevance for preoperative risk assessment. In particular, measurement of ventilatory parameters such as the slope of the increase in minute ventilation in relation to carbon dioxide elimination, VE/VCO_2_ slope (Sun et al., 2002). During the last decade, studies have shown that VE/VCO_2_ slope may be a stronger marker for postoperative complications and mortality after lung resection compared to peak VO_2_ (Brunelli et al., 2012). CPET has a pivotal role in preoperative guidelines before lung cancer surgery (Brunelli et al., 2013) and incorporation of both peak VO_2_ and ventilatory efficiency in an algorithm to improve risk stratification in lung cancer resection has been proposed (Salati & Brunelli, 2016) and recently validated (Kristenson et al., 2022). This approach has also been suggested for preoperative risk stratification for patients evaluated for abdominal surgery (Sivakumar et al., 2022) but this has to our knowledge not been evaluated.

Therefore, the purpose of this pilot study was to evaluate if stratification of patients' functional capacity using a combination of peak VO_2_ and ventilatory efficiency could improve preoperative risk assessment in major upper abdominal surgery.

## 2 MATHERIAL AND METHODS

### 2.1 Participants

The study was designed as a single‐center retrospective pilot study including all patients who underwent CPET as part of preoperative risk evaluation before major upper abdominal surgery (esophagus, stomach, pancreas, and liver) at Linköping University Hospital in Sweden in 2008–2021 (Table 2). Ethical permission was granted (DNr 2021‐05603‐01) and written informed consent was waived by the ethics committee.

### 2.2 Cardiopulmonary exercise testing

Cardiopulmonary exercise testing was performed on a bicycle ergometer (eBike Basic, GE Medical Systems, GmbH), aiming at maximal exhaustion after 8–12 min. The workload was chosen individually (based on standard clinical practice, which accounts for self‐reported fitness alongside clinical judgment) with a 5‐min warm‐up phase between 10 and 50 watts and an incremental ramp protocol with a workload increase of 10–20 watts/min. During CPET, patients were monitored with ECG (Marquette CASE 8000, GE Medical Systems) and repeated systolic blood pressure measurements. The Borg rating of perceived exertion (RPE) scale (Ausania et al., 2012; Benington et al., 2019; Brunelli et al., 2012; Brunelli et al., 2013; Dindo et al., 2004; Fernandez et al., 2015; Gläser et al., 2013; Gläser et al., 2010; Kristenson et al., 2022; Medinger et al., 2001; Salati & Brunelli, 2016; Sivakumar et al., 2022; Snowden et al., 2010; Sun et al., 2002; Wasserman et al., 1996) was used to quantify perceived exhaustion, and the Borg CR‐10 scale was used to assess chest pain and dyspnoea. Blood pressure as well as RPE, dyspnea, and chest‐pain ratings were performed every 2–3 min during the test.

Gas exchange and ventilatory variables were measured breath by breath (Jaeger Oxycon Pro or Vyntus CPX; Viasys Healthcare). The system was calibrated before each CPET. Oxygen uptake (VO_2_), carbon dioxide elimination (VCO_2_), and ventilation (VE) were presented as 10‐s means, excluding the breaths with the highest and lowest values. Peak VO_2_ was defined as the average of the two highest consecutive 10‐s mean VO_2_ intervals at or close to the end of the exercise and was presented as absolute values (mL/min) as well as relative values (mL/kg/min and percent of predicted [% predicted]) (Gläser et al., 2010). Maximum achieved workload was presented as peak power (measured in Watt) as well as % predicted peak power (Gläser et al., 2013).

To obtain ventilatory variables (VE/VCO_2_ slope and the nadir of the ventilatory equivalent of carbon dioxide [EqCO_2_]), automated slopes using a commercial software (Sentry Suite 3.10; CareFusion GmbH) were used, and these were manually adjusted if deemed necessary by the reviewer. VE/VCO_2_ slope was defined as the slope of the increase in VE relative to VCO_2_ increase during the linear portion of the curve up until the respiratory compensation point. EqCO_2_ nadir was defined as the lowest (i.e., nadir) value of VE/VCO_2_ during exercise. The VO2 at the anaerobic threshold (AT) was determined manually. We used a combination of the V‐slope method (1st deflection) and evaluation of the ventilatory equivalents of VO_2_ and VCO_2_, where the AT was defined as where VE/VO_2_ started to increase before an increase in VE/VCO_2_(Levett et al., 2018).

First, patients were grouped based on previously suggested thresholds into either a low or high‐risk group, according to peak VO_2_ (low risk: ≥14, high risk: <14), VO_2_ at AT (low risk: ≥11, high risk: <11), and VE/VCO_2_ slope (low risk: <39, high risk: ≥39). Second, patients were grouped into three risk groups applying a joint assessment of peak VO_2_ and VE/VCO_2_ slope as Group 1 (low risk): peak VO_2_ ≥ 20 mL/kg/min, Group 2 (intermediate risk): peak VO_2_ < 20 mL/kg/min and VE/VCO_2_ slope < 39, and Group 3 (high risk): peak VO_2_ < 20 mL/kg/min and VE/VCO_2_ slope ≥ 39. Patients' comorbidities (coronary artery disease, current treatment for heart failure, current treatment for arrythmia, valvular disease, current treatment for hypertension, previous cerebrovascular insult, chronic obstructive pulmonary disease, chronic kidney failure, or diabetes mellitus) were determined by retrospective journal evaluation and followed international recommendations for use of terminology (Fernandez et al., 2015).

### 2.3 Outcome definitions

The primary outcome was any major cardiopulmonary complication following surgery from admittance to discharge, further defined in Table 1.

**TABLE 1:** Definition of study primary outcome.

The primary outcome was any major cardiopulmonary complication following surgery until discharge and included either of
(a) A major adverse cardiovascular event^a^
• cardiac death
• cerebrovascular death
• non‐fatal cardiac arrest
• acute myocardial infarction
• congestive heart failure
• new cardiac arrhythmia
• angina, or stroke
(b) A major postoperative pulmonary complication^b^
• Pneumonia (patient has received antibiotics for a suspected respiratory infection and met one or more of the following criteria: new or changed sputum, new or changed lung opacities, fever, white blood cell count <4 × 10^9^/L or > 12 × 10^9^/L)
• Moderate respiratory failure (hypoxia requiring continuous positive airway pressure, non‐invasive ventilation, high‐flow nasal cannula or intubation)
• Acute respiratory distress syndrome defined by the Berlin criteria^c^
• Atelectasis recurring bronchoscopy
(c) Pulmonary embolism (verified with computed tomography pulmonary angiography)

^a^ Defined as suggested by Sabaté et al. (2011).

^b^ Modified by the definition of Briez et al. (2012).

^c^ Defined as suggested by ARDS Definition Task Force et al. (2012).

Secondary outcomes were Clavien‐Dindo complications > grade 2 (complications requiring surgical, endoscopic or radiological intervention with or without general anesthesia, life‐threatening complications that require intensive care or death of the patient; Dindo et al., 2004), length of hospital stay, and 90 day mortality.

### 2.4 Statistical analysis

Statistical analysis was performed using SPSS 27.0.0.0 (IBM‐SPSS Inc.). Due to the low number of observations, non‐parametrical statistics were used. Median values were presented with corresponding interquartile range (IQR) and compared with the independent‐samples Mann–Whitney *U* test and frequencies were compared with Fischer's exact test. All tests were two‐sided, and the significance level was set at *p* < 0.05.

## 3 RESULTS

**FIGURE 1:**
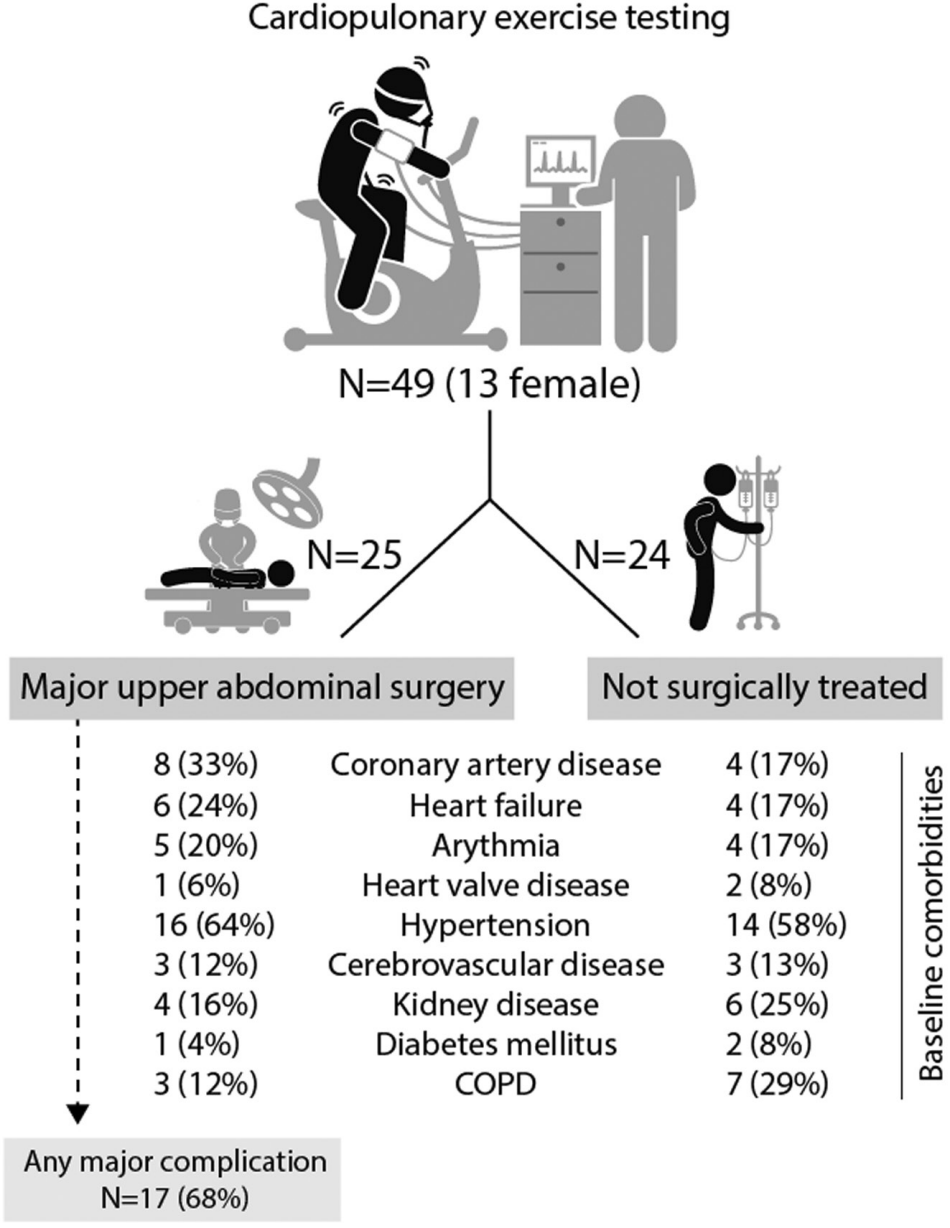
Flowchart of study.

In total, 49 patients were included, as they had performed a preoperative CPET before the decision regarding if the patient would pursue to major upper abdominal surgery or not (Figure 1). The median age was 73 years (range 43–88 years, IQR 68–79), and 74% were men (*n* = 36). Patients were included due to cancer in the esophagus (*n* = 18), stomach (*n* = 6), pancreas (*n* = 16), or liver (*n* = 9).

### 3.1 CPET in patient selected for operation versus not selected for operation

Twenty‐five of the 49 patients were selected for operation due to cancer in esophagus (*n* = 10), stomach (*n* = 4), pancreas (*n* = 10), and liver (*n* = 1). In patients selected for operation, the median age was 73 years (IQR 67–80), and 68% were men (*n* = 17). The median values and IQR for peak VO_2_, AT, and VE/VCO_2_ slope were 18.5 (16.0–22.7) mL/kg/min, 12.8 (11.2–15.6) mL/kg/min, and 30.8 (29.0–37.4).

No statistically significant differences were found in median age (73.0 vs. 73.5, *p* = 0.67) or in presence of comorbidities between patients selected for operation compared to the non‐operated group (Figure 1 and Appendix 1). However, in general, patients not selected for operation had less favorable CPET data including significantly lower values of peak power and peak VO_2_ and higher VE/VCO_2_ slope (Appendix 2).

### 3.2 CPET in patients with versus without complications

In total, the frequency of major cardiopulmonary complications or a complication, according to Clavien–Dindo, was 32% (*N* = 8) and 48% (*N* = 12), respectively. No patient died within 90 days after surgery. The median length of stay was 11 days (IQR 8–22).

No differences were found in presence of comorbidities between patients who suffered a major cardiopulmonary complication compared to those who did not (Table 2). Also, when comparing the median values of preoperative CPET measures, no differences were found in peak power, peak VO_2_, or VO_2_ at AT for patients who suffered a major cardiopulmonary complication compared to those who did not. In contrast, higher (less favorable) values of VE/VCO_2_ slope and EqCO_2_ nadir were present in patients who suffered a major cardiopulmonary complication (Table 3). When analyzing patients who did or did not suffer a complication > grade 2 according to the Clavien‐Dindo classification, lower (less favorable) values were found for % predicted peak power and % predicted peak VO_2_ in patients who experienced a complication (Table 4).

**TABLE 2:** Distribution of gender, comorbidities, and anthropometrics for patients with or without postoperative major cardiopulmonary complications.

	No cardiopulmonary complication		Cardiopulmonary complication		
	*N*	%	*N*	%	*p*
Gender					
Women	6	35	2	25	0.61
Men	11	65	6	75	
Comorbidity					
Coronary artery disease	6	35	2	25	0.61
Heart failure	4	24	2	25	0.94
Arrythmia	4	24	1	13	0.52
Valvular disease	1	6	0	0	0.48
Hypertension	9	53	7	88	0.09
Cerebrovascular insult	1	6	2	25	0.17
COPD	3	18	1	13	0.74
Kidney failure	1	6	0	0	0.48
Diabetes mellitus	1	6	2	25	0.17

Anthropometrics		Median (IQR)		Median (IQR)	*p*
Height, cm	17	172 (165–177)	8	175 (169–183)	0.45
Weight, kg	17	73 (64–85)	8	82 (74–100)	0.09
Body mass index, kg/m^2^	17	24.9 (23.4–27.3)	8	25.7 (24.3–34.3)	0.17

Abbreviations: COPD, Chronic obstructive pulmonary disease; IQR, interquartile range; *p*, Fischer's exact test.

**TABLE 3:** Results from preoperative cardiopulmonary exercise testing for patients with or without postoperative major cardiopulmonary complications.

	Total			No cardiopulmonary complication			Cardiopulmonary complication			*p*
	*N*	Median	IQR	*N*	Median	IQR	*N*	Median	IQR	
Peak power, watt	25	100.0	81.0–132.0	17	100.0	81.0–128.0	8	98.0	68.8–145.3	0.88
% predicted peak Power, %	25	68	55–92	17	68	58–90	8	68	51–92	0.68
Peak VO_2_, mL/min	25	1356	1140–1833	17	1315	1140–1833	8	1599	1121–1855	0.56
Peak VO_2_, mL/kg/min	25	18.5	15.3–22.4	17	18.5	16.0–23.3	8	18.7	12.9–20.7	0.56
% predicted peak VO_2,_ %	25	91	70–104	17	92	71–105	8	84	70–99	0.60
VO_2_ at AT, mL/kg/min	24	12.8	11.2–15.6	17	13.2	11.5–15.8	7	12.1	9.1–15.2	0.30
VE/VCO_2_ slope	25	30.8	29.0–37.4	17	29.7	28.6–34.9	8	37.3	31.1–45.1	0.03
EqCO_2_ nadir	25	30.8	27.9–34.7	17	29.1	27.6–33.6	8	35.0	31.1–40.1	0.048

Abbreviations: EqCO_2_, ventilatory equivalent for carbon dioxide; ICR, interquartile range; *p*, Independent‐Samples Mann–Whitney *U* Test; VCO_2_, carbon dioxide elimination; VE, minute ventilation; VO_2_peak, peak oxygen uptake.

**TABLE 4:** Results from preoperative cardiopulmonary exercise test for patients with or without any postoperative complication according to Clavien–Dindo > grade 2.

	Postoperative complication			No postoperative complication			
	*N*	Median	IQR	*N*	Median	IQR	*p*
Peak power, Watt	12	90.5	80.8–114	13	113	71.5–142	0.40
% predicted peak power, %	12	62	54–75	13	85	60.5–100	0.04
Peak VO_2_, mL/min	12	1354	1122–1655	13	1682	1092–1961	0.44
Peak VO_2_, mL/kg/min	12	16.6	14.9–19.7	13	21.8	16.0–25.8	0.01
% predicted peak VO_2,_ %	12	82	66–92	13	104	81.108	0.03
VO_2_ at AT, mL/kg/min	11	12.1	11.4–14.2	13	14.4	10.7–17.0	0.25
VE/VCO_2_ slope	12	32.8	29.4–41.7	13	30.5	27.5–34.9	0.26
EqCO_2_ nadir	12	32.7	29.5–37.9	13	28.7	25.7–33.4	0.05

Abbreviations: EqCO_2_, ventilatory equivalent for carbon dioxide; ICR, interquartile range; *p*, Independent‐Samples Mann–Whitney *U* Test for comparison between patients who sustained versus did not sustain a postoperative complication; VO_2_peak, peak oxygen uptake; VCO_2_, carbon dioxide elimination; VE, minute ventilation.

#### 3.2.1 Risk stratification

**FIGURE 2:**
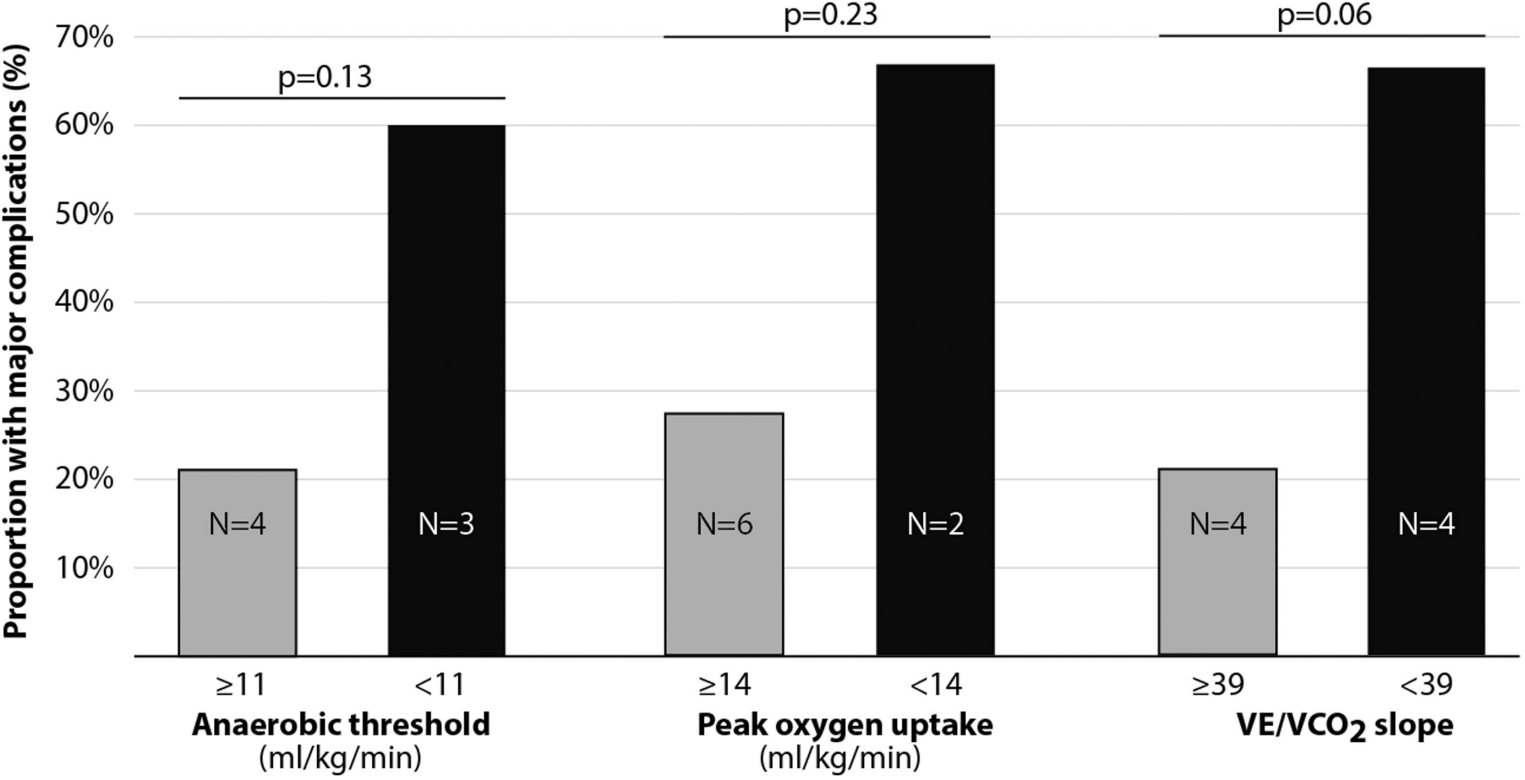
Proportion of patients who suffered or did not suffer a major cardiopulmonary complication after surgery stratified by traditional thresholds for measures from the preoperative cardiopulmonary exercise test. Frequencies of complications were compared with Fischer's exact test.

**FIGURE 3:**
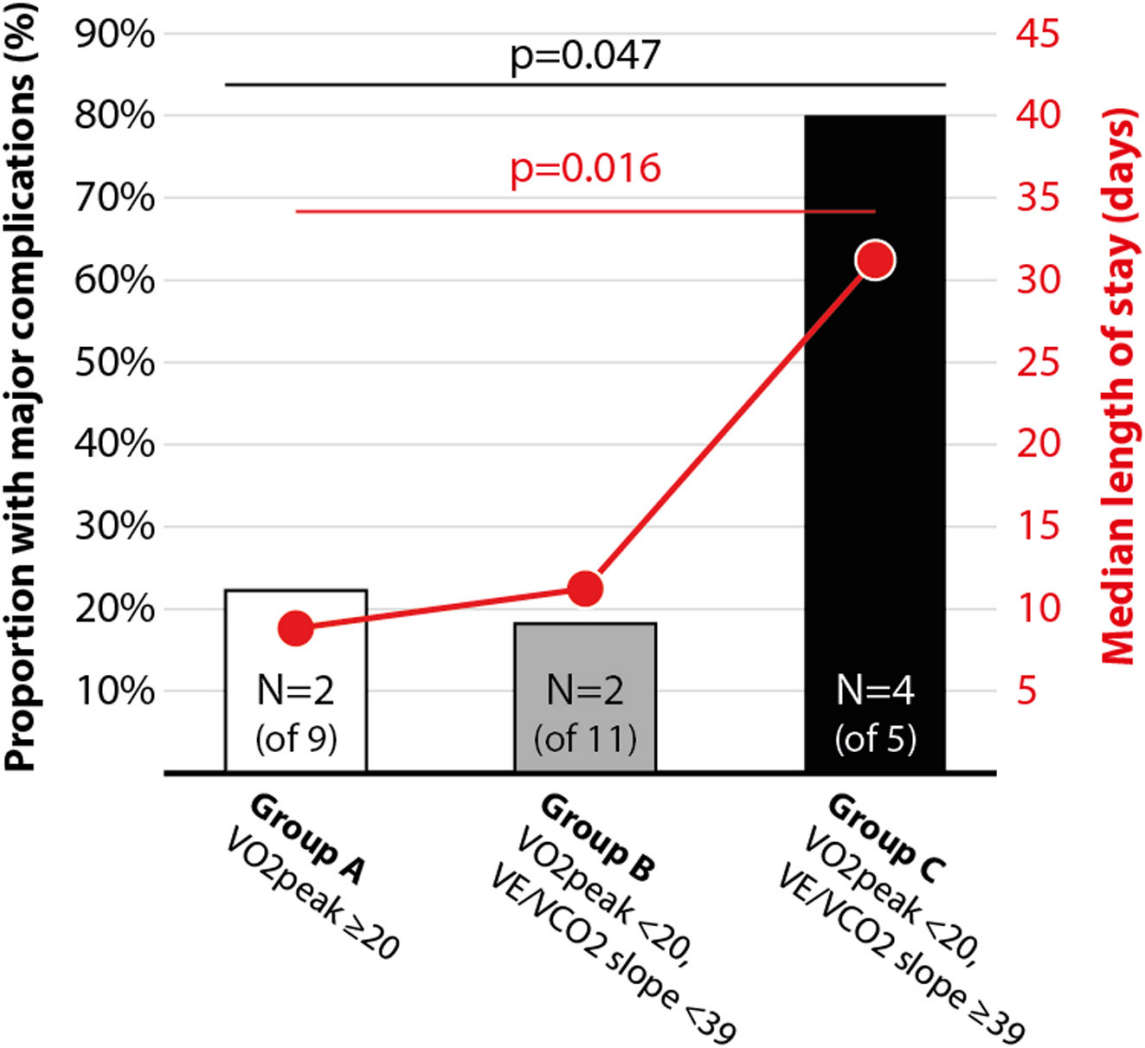
Proportion of patients who suffered or did not suffer a major cardiopulmonary complication after surgery and length of hospital stay for patients from different risk groups based on VO_2_peak (peak oxygen consumption) measured in mL/kg/min, and VE/VCO_2_‐slope (ventilatory efficiency) from the preoperative cardiopulmonary exercise test. Median values of length of stay were compared with the Independent‐Samples Mann–Whitney *U* Test and frequencies of complications were compared with Fischer's Exact Test.

There were statistically non‐significant trends that patients with low peak VO_2_ or VO_2_ at AT or high VE/VCO_2_ slope values (defined by previously suggested thresholds) had an increased frequency of complications (Figure 2). However, when using a combined stratification by peak VO_2_ and VE/VCO_2_ slope, patients with peak VO_2_ < 20 mL/kg/min and VE/VCO_2_ slope ≥ 39 (group 3, higher risk) had a statistically significant higher rate of major cardiopulmonary complications and longer length of stay compared to the other risk groups (Figure 3).

## 4 DISCUSSION

In this pilot study of patients who performed CPET before major upper abdominal surgery, we found that patients who suffered a major cardiopulmonary complication had significantly higher (less favorable) values of ventilatory efficiency compared to those who did not sustain a complication. Importantly, we found that by using a combination of low aerobic capacity (peak VO_2_ < 20 mL/kg/min) and ventilatory efficiency (VE/VCO_2_‐slope ≥ 39), we were able to identify a group of patients with a particularly high frequency of complications (80%).

Of note, we did not find any significant differences in age or in prevalence of comorbidities in patients selected versus those not selected for surgery. However, patients not selected for surgery were found to have a lower functional capacity, reflected by less favorable results on multiple CPET measures (lower aerobic capacity, ventilatory efficiency as well as anaerobic threshold). When analyzing the risk of postoperative cardiopulmonary complications, again, no differences were found in prevalence of comorbidities between patients who suffered a complication compared to those who did not. However, when comparing the median values of preoperative CPET measures, higher (less favorable) values of VE/VCO_2_ slope and EqCO_2_ nadir were present in patients who suffered a major cardiopulmonary complication. Interestingly, no differences were found in the more traditional measures peak power, peak VO_2_, or VO_2_ at AT for patients who suffered a major cardiopulmonary complication compared to those who did not. These results harmonize with the results from a large multicenter study where the thresholds peak VO_2_ 14 mL/kg/min and VO_2_ at AT 11 mL/kg/min were not significantly related to an increased risk for the primary outcome (death or myocardial infarction within 30 days after surgery; Wijeysundera et al., 2018). This stresses the importance in using relevant measures and thresholds in preoperative CPET studies.

Interestingly, the strongest risk prediction was found when combining the two measures peak VO_2_ and VE/VCO_2_‐slope. This has been evaluated with promising results in thoracic surgery (Kristenson et al., 2022), but this is, to our knowledge, the first study adopting this approach in risk stratifications studies within major abdominal surgery.

### 4.1 Ventilatory efficiency

Ideally, in the lung, there is a perfect match between perfusion and ventilation. When a mismatch occurs, gas exchange is impaired, and a greater ventilation is requiring for a given output of CO_2_. This ventilatory inefficiency (most often due to increased dead space ventilation) is reflected as an increase in VE/VCO_2_ slope measured during CPET (Sun et al., 2002). For example, a VE/VCO_2_ slope value of 39 means that a patient needs to exhale 39 liters of air to eliminate 1 liter of CO_2_. VE/VCO_2_ slope has in recent decades emerged as a tool to assess both the presence and severity of heart or lung disease (Medinger et al., 2001; Wasserman et al., 1996).

VE/VCO_2_ slope determination was first used by cardiologists evaluating patients with heart failure (Sun et al., 2002). Therefore, most studies using CPET for preoperative risk stratification refer to thresholds of VE/VCO_2_ slope generated from historical data in heart failure patients (Chua et al., 1997; Corrà et al., 2002), most often using a cutoff of 35 to identify high‐risk patients (Brunelli et al., 2012; Shafiek et al., 2016). Recent studies in major abdominal surgery have identified patients with a VE/VCO_2_ slope ≥ 39 to have an increased risk of mortality (Wilson et al., 2019) and this threshold was therefore used in the current study. However, as previous authors have suggested, using a single threshold entails a binary approach toward risk assessment, which is problematic in the real, more complex practice of preoperative CPET (Older, 2013; Sivakumar et al., 2022) Therefore, future studies in larger cohorts should strive to identify multiple thresholds that privilege sensitivity and specificity separately (Wilson, 2018).

### 4.2 Clinical implication

Cardiopulmonary exercise testing has several advantages compared to other means of assessing functional capacity. First, it is possible to determine whether the test was at maximal effort by the patent, which is essential if maximum functional capacity is to be evaluated (such as peak VO_2_). Second, during CPET, several other variables of importance than maximum capacity can be assessed, such as signs of coronary artery disease, pulmonary comorbidities or ventilatory inefficiency. Third, several of these variables, including VE/VCO_2_ slope is measured at submaximal effort and thus does not require a truly maximal test. Future studies should focus on which patients that can be assessed by screening with a more widely available functional test, and which patients benefit from the more comprehensive CPET (Junttila et al., 2022).

After having identified patients at particularly high risk for major cardiopulmonary complications, how can the perioperative physician translate these results to clinical decision‐making, ultimately decreasing the risk for the individual patient? First, prehabilitation can be initiated which has been shown to increase functional capacity and lower the risk of complications and mortality for patients undergoing abdominal surgery (Pang et al., 2022; Zarate Rodriguez et al., 2023). Of note, exercise training has been shown to increase not only VO_2_ peak, but also ventilatory efficiency (i.e., lowering VE/VCO_2_ slope) for patients with heart failure or pulmonary hypertension (Mehani & Abdeen, 2017). It remains to be evaluated whether preoperative risk defined by the combination of peak VO_2_ and VE/VCO_2_ slope can be affected by prehabilitation in abdominal surgery. Second, if a previously unknown pathology is identified, treatment can be initiated to treat the underlying condition. Third, the data derived from CPET may be used to inform collaborative decision‐making and contribute to preoperative risk assessment (Levett et al., 2018). Fourth, high‐risk patients that proceed to operation should be assessed and evaluated with caution to identify complications before severe organ failure occurs, a situation that has been called the “failure of rescue” (Ghaferi et al., 2009). Previous studies in colorectal patients have showed that patients preoperatively identified as having an intermediary risk for postoperative complications, the risk was dependent on whether they were treated on a high dependency unit or a standard postoperative ward (Swart et al., 2017). This could in turn speak in favor of having different postoperative care or readiness for complications depending on preoperative risk assessment, where CPET may play an important role.

### 4.3 Limitations

This is a retrospective, single center pilot study with the aim of exploring if strategies for risk stratification used in thoracic surgery also could be applied in a major upper abdominal surgery cohort. Thus, the total sample is small, and the results should therefore be interpretated with caution. The low power did not allow for any adjustment for other preoperative comorbidities, although there were no statistically significant differences in frequencies of comorbidities between patients who sustained a major cardiopulmonary complication compared to those who did not. The small sample size also precluded stratification by the different types of cancers included, which should be considered in future studies including more patients.

## 5 CONCLUSION

Patients who suffered a major cardiopulmonary complication following major upper abdominal surgery had significantly lower (worse) ventilatory efficiency at preoperative CPET compared to those who did not. Having a low ventilatory efficiency in combination with a low aerobic capacity was associated with a particularly high risk (80%) of suffering a major cardiopulmonary complication. The results from this pilot study calls for validation in larger studies in order to further improve risk assessment in this group of patients.

### AUTHOR CONTRIBUTION

KK is the guarantor of the study. EG performed the data collection for the study. KK and EG performed data analyses. KK and EG drafted the first version of the manuscript. All authors listed have provided substantial contributions to the conception, design, data acquisition, analysis, and interpretation of this work, and all authors participated in revising the manuscript after critical review. All authors approved the final version of the manuscript.

### CONFLICT OF INTEREST STATEMENT

The authors declare that they have no conflict of interest.

## APPENDIX 1 DISTRIBUTION OF GENDER, COMORBIDITIES AND ANTHROPOMETRICS AMONG PATIENTS WHO WERE AND WERE NOT SELECTED FOR OPERATION.

### 1.1

**Table:**

	Total		Not selected for surgery		Selected for surgery		
	*N*	%	*N*	%	*N*	%	*p*
Gender							
Women	13	27	5	21	8	32	0.52
Men	36	73	19	79	17	68	
Comorbidity							
Coronary artery disease	12	25	4	17	8	33	0.32
Heart failure	10	20	4	17	6	24	0.73
Arrythmia	9	18	4	17	5	20	1.00
Valvular disease	3	6	2	8	1	6	0.61
Hypertension	30	61	14	58	16	64	0.77
Cerebrovascular insult	6	12	3	13	3	12	1.00
COPD	10	20	6	25	4	16	0.50
Kidney failure	3	6	2	8	1	4	0.61
Diabetes mellitus	10	20	7	29	3	12	0.17

Anthropometrics		Median (IQR)		Median (IQR)		Median (IQR)	*p*
Height, cm	49	172 (166–178)	24	172 (164–179)	25	174 (166–178)	0.84
Weight, kg	49	73 (65–84)	24	70 (62–81)	25	75 (68–87)	0.16
Body mass index, kg/m^2^	49	24.6 (23.4–28.4)	24	24.1 (22.1–28.1)	25	25.2 (23.8–30.5)	0.24

Abbreviations: COPD, Chronic obstructive pulmonary disease; *p*, Fischer's exact test; IQR, interquartile range.

## APPENDIX 2 RESULTS FROM PREOPERATIVE CARDIOPULMONARY EXERCISE TEST FOR PATIENTS WHO WERE AND WERE NOT SELECTED FOR OPERATION.

### 2.1

**Table:**

	Total			Not selected for surgery			Selected for surgery			
	*N*	Median	IQR	*N*	Median	IQR	*N*	Median	IQR	*p*
Peak power, watt	49	85.0	68.0–115	24	74.0	61.8–90.0	25	100.0	81.0–132.0	0.007
% predicted peak power, (%)	49	59	46–82	24	50	38–62	25	68	55–92	0.002
Peak VO_2_, ml/min	49	1240	996–1697	24	1100	931–1321	25	1356	1140–1833	0.006
Peak VO_2_, ml/kg/min	49	17.1	14.1–20.6	24	15.5	12.9–19.7	25	18.5	15.3–22.4	0.006
% predicted peak VO_2_, (%)	49	77	58–96	24	60	51–88	25	91	70–104	0.002
VO_2_ at AT, ml/kg/min	45	12.6	11.0–15.1	21	12.5	10.0–14.5	24	12.8	11.2–15.6	0.35
VE/VCO_2_ slope	46	34.4	29.7–41.2	21	40.1	32.9–43.0	25	30.8	29.0–37.4	0.02
EqCO_2_ nadir	47	32.7	28.8–37.6	22	34.8	30.2–39.3	25	30.8	27.9–34.1	0.58

Abbreviations: EqCO_2_, ventilatory equivalent for carbon dioxide; ICR, interquartile range; *p*, Independent‐Samples Mann–Whitney *U* Test for comparison between patients who sustained versus did not sustain a postoperative complication; VO_2_peak, peak oxygen uptake; VCO_2_, carbon dioxide elimination; VE, minute ventilation.
